# Hepcidin, Serum Iron, and Transferrin Saturation in Full-Term and Premature Infants during the First Month of Life: A State-of-the-Art Review of Existing Evidence in Humans

**DOI:** 10.1093/cdn/nzaa104

**Published:** 2020-06-17

**Authors:** James H Cross, Andrew M Prentice, Carla Cerami

**Affiliations:** Epidemiology and Population Health, Medical Research Council Unit The Gambia at the London School of Hygiene & Tropical Medicine, Fajara, Banjul, The Gambia; Epidemiology and Population Health, Medical Research Council Unit The Gambia at the London School of Hygiene & Tropical Medicine, Fajara, Banjul, The Gambia; Epidemiology and Population Health, Medical Research Council Unit The Gambia at the London School of Hygiene & Tropical Medicine, Fajara, Banjul, The Gambia

**Keywords:** nutritional immunity, host–pathogen interaction, hepcidin, neonates, hypoferremia, transferrin, serum iron

## Abstract

Neonates regulate iron at birth and in early postnatal life. We reviewed literature from PubMed and Ovid Medline containing data on umbilical cord and venous blood concentrations of hepcidin and iron, and transferrin saturation (TSAT), in human neonates from 0 to 1 mo of age. Data from 59 studies were used to create reference ranges for hepcidin, iron, and TSAT for full-term-birth (FTB) neonates over the first month of life. In FTB neonates, venous hepcidin increases 100% over the first month of life (to reach 61.1 ng/mL; 95% CI: 20.1, 102.0 ng/mL) compared with umbilical cord blood (29.7 ng/mL; 95% CI: 21.1, 38.3 ng/mL). Cord blood has a high concentration of serum iron (28.4 μmol/L; 95% CI: 26.0, 31.1 μmol/L) and levels of TSAT (51.7%; 95% CI: 46.5%, 56.9%). After a short-lived immediate postnatal hypoferremia, iron and TSAT rebounded to approximately half the levels in the cord by the end of the first month. There were insufficient data to formulate reference ranges for preterm neonates.

## Introduction

### Iron homeostasis during pregnancy

**FIGURE 1:**
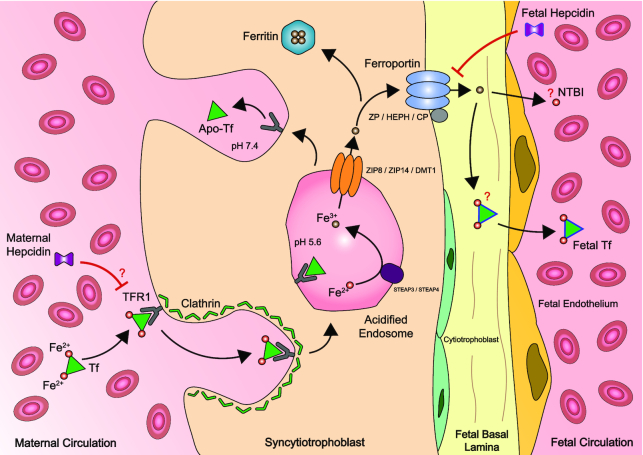
Placental iron transfer between mother and fetus. Syncytiotrophoblasts in the placental villi take up Tf-bound iron from the maternal circulation by endocytosis via TFR1. Iron is released from TFR1 in acidified endosomes and transferred into the syncytiotrophoblast cytoplasm. Ferroportin transports iron out of placental syncytiotrophoblasts, and then ceruloplasmin, hephaestin, and zyklopen oxidize Fe^2+^ to Fe^3+^, helping it pass through the endothelium to reach the fetal circulation. It is still unclear as to whether newly transported iron enters the fetal circulation as NTBI or bound to fetal Tf. Fetal-derived hepcidin is believed to regulate ferroportin expression on the fetal basal side of placental syncytiotrophoblasts (12, 25). Maternal-derived hepcidin is believed to play a role in regulating TFR1 expression on the maternal side of placental syncytiotrophoblasts (31). Apo-Tf, unsaturated transferrin; CP, ceruloplasmin; DMT1, divalent metal transporter 1; fetal Tf, fetal-derived transferrin; Fe^2+^, ferrous iron; Fe^3+^, ferric iron; HEPH, hephaestin; NTBI, non-transferrin-bound iron; Tf, transferrin; TFR1, transferrin receptor 1; STEAP, six-transmembrane epithelial antigen of prostate; ZIP, zinc and iron related protein; ZP, zyklopen.

Three important mediators of hepcidin synthesis—iron status, inflammation, and erythropoiesis—are all altered during pregnancy (1–4). Iron demand on the mother increases significantly to support expanded maternal erythropoiesis and iron requirements of the growing fetus (5–9). During pregnancy, the placenta transfers ∼270 mg Fe from the mother to the fetus via the placenta (10, 11). Syncytiotrophoblasts in the placental villi take up transferrin-bound iron from the maternal circulation by endocytosis via transferrin receptor 1 (TFR1) (**Figure 1**) (12, 13). As reviewed in Cao and Fleming (14) and Fisher and Nemeth (15), iron is released from TFR1 and transferred from the acidified endosome into the syncytiotrophoblast cytoplasm by divalent metal transporter 1 (13), zinc and iron related protein 8 (ZIP8) (16), and zinc and iron related protein 14 (ZIP14) (17). Ferroportin transports iron out of placental syncytiotrophoblasts, and then ceruloplasmin, hephaestin, and zyklopen assist in the oxidization of Fe^2+^ to Fe^3+^, helping it pass through the endothelium to reach the fetal circulation (18–20).

### Maternal control of fetal and early neonatal iron metabolism

Increases in maternal dietary iron uptake and placental iron transfer occur in the second and third trimesters (21, 22), when maternal hepcidin decreases to trigger increased duodenal iron absorption (23), splenic macrophage iron recycling, and the release of maternal hepatic iron stores (24–26). The resulting increased circulating maternal iron is then freely available for transfer to the fetus. Factors that are thought to contribute to maternal hepcidin suppression in the second and third trimesters include maternal iron deficiency, erythropoiesis in the mother or fetus (25), estrogen (26), and progesterone receptor membrane component-1 (27). Conflicting evidence now exists as to whether pregnancy-induced plasma dilution may also play a role (15, 28).

### Fetal control of fetal and early neonatal iron metabolism

Eighty percent of all the iron transference from the mother to the fetus occurs in the last trimester (29). An illustration of the fetal demand for iron [amounting to 1.6–2.0 mg · kg^−1^ · d^−1^ (30)] is that umbilical cord

blood contains a higher serum iron concentration than in the maternal circulation and at delivery infants have higher total body iron per kilogram than that measured in their mothers or in healthy adults (32–43). This pattern is seen even in anemic mothers and their infants (30, 42, 44, 45). The relative roles of maternal and fetal hepcidin concentrations in controlling placental iron transport are unclear and may change during the course of gestation (24, 28, 41, 43, 44, 46–54). As iron becomes more available in the last months of pregnancy, the fetus synthesizes hepcidin probably to control the rate of placental iron transfer and thereby to protect itself from iron-overload (15, 28, 55). Evidence showing the importance of fetal hepcidin includes: *1*) umbilical cord hepcidin concentrations at birth are higher than maternal concentrations before and during delivery (24, 46, 43, 53, 56, 57); and *2*) in pregnancies with multiple gestations, differences in cord hepcidin between siblings explained a greater fraction of variability in cord hemoglobin, serum ferritin, soluble transferrin receptor, and erythropoietin than maternal hepcidin concentrations (49).

### Placental control of fetal and early neonatal iron metabolism

The placenta may also independently regulate iron transfer to the fetus in some scenarios (58). A reduction of ferroportin expression on the apical fetal-facing membrane of placental syncytiotrophoblasts during maternal iron deficiency, in addition to increased expression of TFR1 on the maternal-facing side supports this hypothesis (28). Sangkhae et al. (28) propose that during maternal iron deficiency, iron is held in the placenta to ensure that its metabolic homeostasis is maintained. Placental protein synthesis and critical transfer mechanisms can then continue, ensuring the more detrimental condition of placental dysfunction does not occur. These findings were observed in murine and in vivo human trophoblast models, but not in respect to the human pregnancies analyzed (28).

### Impact of labor and delivery on hepcidin

Childbirth is an intensely stressful event. Inflammatory pathways (including IL-6-mediated pathways) are induced at the onset of human labor, even in the absence of intrauterine infection (59–66). Initiating stimuli for IL-6 production and release could involve the endocrine events of labor (65–67), mechanical distension of the membranes and cervix (smooth muscle) (59, 67–70), placental hypoxia and/or hypo-perfusion (67, 71), fetal hypoxia-acidemia (72), pain (73), or exposure to infective agents (64, 66, 67, 74). The production of IL-6 leads to an increase in hepcidin concentrations along with a massive influx of immune cells (predominantly neutrophils) into the cervix, decidua, myometrium, chorioamnionic membranes, and amniotic fluid (65, 75). This further exacerbates the rise in IL-6 and other cytokines (73, 76). The increase in postdelivery maternal hepcidin concentrations is larger with cesarean deliveries (550% increase) than with standard vaginal deliveries (300% increase) (77). This is most likely due to the surgical procedure and the subsequent inflammation. Similar increases in serum hepcidin are seen postoperatively during other abdominal surgeries (78). The effect of this maternal rise in hepcidin before, during, and immediately after childbirth on the late fetal/early neonatal iron status is unknown, although like IL-6 (79), hepcidin is not thought to cross the placenta (80).

### Effects of infection on neonatal serum hepcidin concentrations

Intra-amniotic infections can cause an increase in fetal hepcidin (81). Multiple studies have documented an association of chorioamnionitis, perinatal acidosis, and neonatal sepsis with high umbilical cord hepcidin concentrations (81–86). For example, an extremely high cord hepcidin concentration (437.6 ng/mL) was found in a neonate with confirmed *Enterococcus faecalis* early-onset sepsis (84). Similarly, very-low-birth-weight, premature neonates with late-onset culture-confirmed sepsis exhibit elevated concentrations of hepcidin (83). Nevertheless, despite the well-documented regulatory pathways of infection and inflammation on iron regulation, it is important to note that multiple publications have shown a lack of correlation between hepcidin, IL-6, and C-reactive protein (CRP) in sick neonates (84, 87). This is likely due to differences in the biochemical kinetics of these molecules. IL-6 concentrations spike very early in the course of perinatal infection, whereas the rise of CRP is delayed.

### Standardizing hepcidin measurements

Multiple assays, including MS and immunochemistry ELISA methods, are available to quantify hepcidin in various body fluids (urine, serum, and plasma) (88). However, in the studies included in this state-of-the-art review, none of these methods were calibrated using the same standards and, as a result, there were significant differences in hepcidin values between studies (89, 90).

In 2016, van der Vorm et al. (89) harmonized many of the available hepcidin ELISA assays using native, lyophilized plasma with cryolyoprotectant as a commutable candidate reference material. Linear equations were formulated to standardize the hepcidin assays (89). These equations can now be used to conduct post hoc standardization of noncalibrated test results, aiding the retrospective comparison of data from previous publications. We have used these equations in this state-of-the-art review to generate standardized hepcidin values (**Supplemental Table 1**). Standardized reference material, which was refined in 2019, is now available for purchase, allowing hepcidin measurements to be standardized in all laboratories (90).

To our knowledge, this is the first time that retrospective comparisons have been made between serum hepcidin concentrations in different studies, using post hoc standardized values to calculate weighted mean averages in umbilical cord and venous blood.

This state-of-the-art review contributes this comparative analysis and also offers an example for how other authors could approach retrospective comparisons of hepcidin concentrations from different studies.

## Methods

**FIGURE 2:**
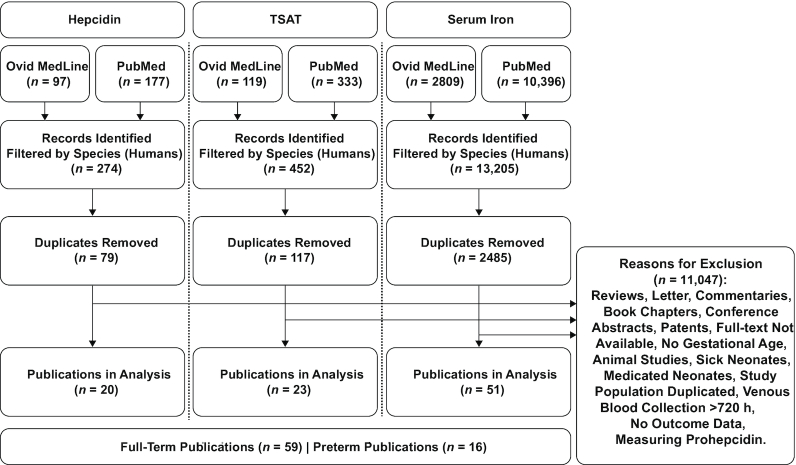
Flow diagram of the literature search and selection criteria for retrieving publications on hepcidin, TSAT, or serum iron in neonates over the first month of life. TSAT, transferrin saturation.

**FIGURE 3:**
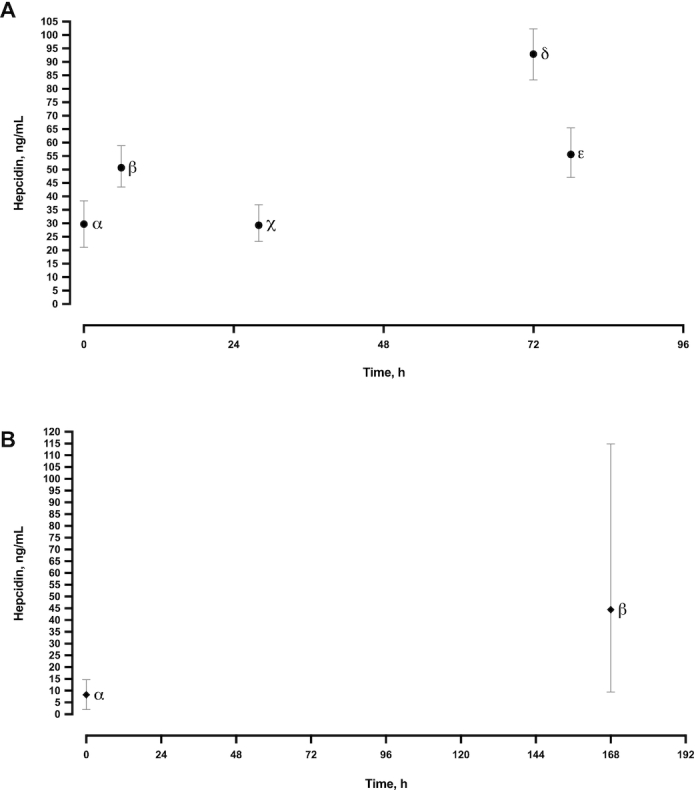
Standardized hepcidin over the neonatal period. (A) Full-term neonates: α shows the weighted mean (95% CI) for all studies seen in Supplemental Figure 1A; β, χ, and ε show Prentice et al. (99); δ shows Kulik-Rechberger et al. (46). (B) Preterm neonates: α shows the weighted mean (95% CI) for all studies seen in Supplemental Figure 1B; β shows Uijterschout et al. (100).

**FIGURE 4:**
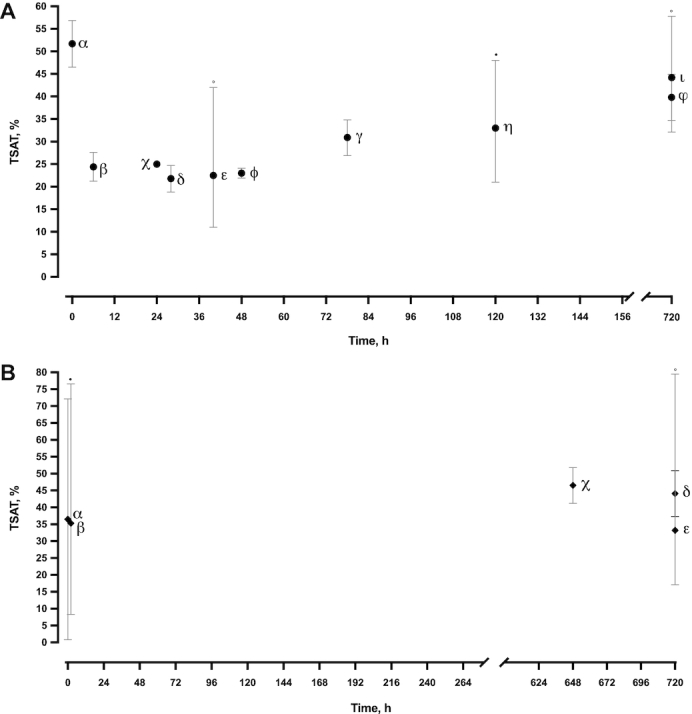
Transferrin saturation over the neonatal period. (A) Full-term neonates: α shows the weighted mean (95% CI) for all studies seen in Supplemental Figure 2A; β, γ, and δ show Prentice et al. (99); χ shows Al-Tawil et al. (102); ε shows Balogh et al. (94); ϕ shows Andersson et al. (103); η shows Milman et al. (39); ι shows Kitajima et al. (104); φ shows Yamada and Leone (105). (B) Preterm neonates: α shows the weighted mean (95% CI) for all studies seen in Supplemental Figure 2B; β shows Lackmann et al. (106); χ shows Celik et al. (107); δ shows Yamada and Leone (105); ε shows Kitajima et al. (104). All values are means (95% CIs), unless marked with ° [median (range)] or • [median (95% CI)]. Lackmann et al. (106) (β) data from the 3 study groups (<32 wk, 33–34 wk, and 35–36 wk) were averaged because all groups are classed as preterm neonates and were bled at the same time of life. TSAT, transferrin saturation.

**FIGURE 5:**
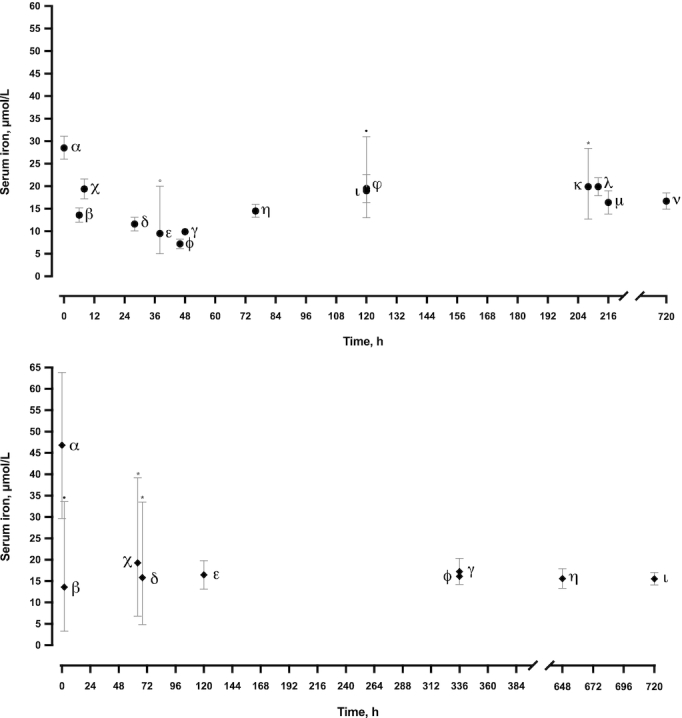
Serum iron over the neonatal period. (A) Full-term neonates: α shows the weighted mean (95% CI) for all studies seen in Supplemental Figure 3A; β, δ, and η show Prentice et al. (99); χ shows Patidar et al. (108); ε shows Balogh et al. (94); ϕ shows Szabó et al. (109); γ shows Andersson et al. (103); ι shows Milman et al. (39); φ shows Tsuzuki et al. (110); κ shows Tiker et al. (111); λ shows Yapakçı et al. (112); μ shows Ozkiraz et al. (113); ν shows Yamada and Leone (105). (B) Preterm neonates: α shows the weighted mean (95% CI) for all studies seen in Supplemental Figure 3B; β shows Lackmann et al. (106); χ and δ show Tiker et al. (111); ε shows Tsuzuki et al. (110); ϕ shows Schiza et al. (114); γ shows Yapakçı et al. (112); η shows Celik et al. (107); ι shows Yamada and Leone (105). All values are mean (95% CI), unless marked with * [mean (range)], ° [median (range)], or • [median (95% CI)]. Lackmann et al. (106) (β) data from the 3 study groups (<32 wk, 33–34 wk, and 35–36 wk) were averaged because all groups are classed as preterm neonates and were bled at the same time of life.

In March 2019, we reviewed the literature searching 2 databases—PubMed and Ovid Medline—with no restrictions on language. The original search was for human studies only published during the date range of 1 January, 1975 to 1 December, 2019. Corresponding authors of extracted publications were not contacted. One individual carried out the inclusion/exclusion process of the retrieved studies, and there was no assessment of bias or the quality of studies as seen in a systematic review process. **Table 1** displays the search strategy used. **Figure 2** shows the flow diagram of the literature search. The search generated publications containing data on cord and venous concentrations of hepcidin and serum iron, and levels of transferrin saturation (TSAT), in the neonatal period. Studies that analyzed healthy neonates were included. Mean, median, or range of the gestational age of the study population was a requirement for inclusion. Neonates ≥37 wk at delivery were regarded as full-term-birth (FTB) neonates. Studies or study groups with a gestational age <37 wk were classed as premature [preterm-birth (PTB)] neonates. Retrieved publications had to report a mean time of bleed 0–720 h postdelivery to be analyzed. Mean (SD or 95% CI) or median (range, IQR, or 95% CI) data were extracted from the included publications. Studies reporting means (95% CIs) were included in the calculation of weighted means (95% CIs) and the associated **Figures 3** –**5**. Reference ranges for adults and children were presented for comparison (91, 92). Many retrieved publications did not stratify results by birth weight; as a result, this variable was not recorded in **Tables 2**–**7**. Publications were not stratified by sample type (serum or plasma) owing to the overall lack of studies. If multiple publications on the same study population were retrieved, only 1 was included in the analysis.

**TABLE 1:** Literature search strategy^1^

Parameter	Database	Search strategy
Hepcidin	Ovid Medline	(Human) AND (neonate OR neonates OR infant OR infants OR baby OR babies OR cord OR “umbilical cord”.mp.) AND (hepcidin OR prohepcidin.mp.)
	PubMed	(Human) AND (neonate OR neonates OR infant OR infants OR baby OR babies OR cord OR “umbilical cord”) AND (hepcidin OR prohepcidin)
TSAT	Ovid Medline	(Human) AND (neonate OR neonates OR infant OR infants OR baby OR babies OR cord OR “umbilical cord”.mp.) AND (“transferrin saturation” OR TSAT.mp.)
	PubMed	(Human) AND (neonate OR neonates OR infant OR infants OR baby OR babies OR cord OR “umbilical cord”) AND (“transferrin saturation” OR TSAT)
Serum iron	Ovid Medline	(Human) AND (neonate OR neonates OR infant OR infants OR baby OR babies OR cord OR “umbilical cord”.mp.) AND (“serum iron” OR iron.mp.)
	PubMed	(Human) AND (neonate OR neonates OR infant OR infants OR baby OR babies OR cord OR “umbilical cord”) AND (“serum iron” OR iron)

^1^Searches conducted via PubMed and Ovid Medline. TSAT, transferrin saturation.

The standardization of hepcidin values generated by different ELISA assays was performed using the slopes and intercepts from van der Vorm et al. (89). This was performed for studies that used ELISA test kits from DRG [Hepcidin-25 (human) enzyme immunoassay (EIA) Kit, DRG], Bachem (Hepcidin-25 EIA Kit, Bachem), and Intrinsic Lifesciences (Intrinsic Hepcidin ELISA Kit, Intrinsic Lifesciences). It was not possible to standardize hepcidin values acquired using the ELISA from Hangzhou Eastbiopharm (Hangzhou Eastbiopharm Co. Ltd.) and mass spectroscopy (MCProt Biotechnology), used in Basu et al. (51) and Ichinomiya et al. (82), respectively. Prohepcidin was not included in the analysis because it is a poor proxy for biochemically active hepcidin-25 (93–98).

The software packages Stata IC version 15 (StataCorp LP) and R (R: A Language and Environment for Statistical Computing, R Foundation for Statistical Computing, 2020) were used to analyze the data. To calculate the CI around the weighted mean, the weighted variance was calculated using the *wtd.var* function from the R package Hmisc. The SE derived from this weighted variance was then used to calculate the *t* statistic (i.e., weighted mean divided by weighted SE), from which the 95% CI was derived. GraphPad Prism version 8 (GraphPad Software) software was used to produce the graphical representation of the results.

## Results

The initial search of 2 electronic databases for 3 different iron markers yielded 13,931 publications. After the exclusion of duplicated studies and selection criteria filtering, 20 publications were included in the analysis for hepcidin, 23 publications for TSAT, and 51 publications for serum iron. Many of these studies were found to contain information on multiple parameters of interest. Overall, we identified 59 publications containing data on hepcidin, serum iron, or TSAT in FTB neonates. Sixteen publications were found to contain data on PTB neonates.

In publications detailing the effects of cord clamping interventions, all retrieved cord blood values were from groups that underwent 60 s of delayed cord clamping. This is consistent with current WHO policy (101). Cord blood weighted mean values are shown in Tables 2–7 and are represented by a dashed line in **Supplemental Figures 1–3** and α (95% CI) in Figures 3 –5.

### Hepcidin

Standardized weighted mean umbilical cord blood hepcidin concentrations were higher in FTB neonates (29.7 ng/mL; 95% CI: 21.1, 38.3 ng/mL) than in PTB neonates (8.4 ng/mL; 95% CI: 2.0, 14.7 ng/mL) (Supplemental Figure 1A, 1B, Tables 2, 3). Full-term cord blood hepcidin concentrations were >100% higher than in adult male (13.1 ng/mL; 95% CI: 1.4, 43.2 ng/mL) and female (10.6 ng/mL; 95% CI: 1.4, 43 ng/mL) reference ranges (Table 2). FTB standardized venous hepcidin concentrations increased (61.1 ng/mL; 95% CI: 20.1, 102.0 ng/mL) over the first 4 d of life (Figure 3A). This trend is unclear for PTB neonates owing to the lack of studies (Table 3, Figure 3B). No studies were retrieved that assessed postdelivery venous blood samples >77 h in FTB or >168 h in PTB neonates.

### TSAT

The weighted mean TSAT in cord blood was higher in FTB neonates (51.7%; 95% CI: 46.5%, 56.9%) than in PTB neonates (36.5%; 95% CI: 0.8%, 72.1%) (Tables 4, 5, Supplemental Figure 2). Cord blood TSAT in FTB neonates was double the reference levels found in adults (23.5%; 95% CI: 12%, 38.8%) and children aged 1–5 y (19.4%; 95% CI: 8.2%, 32.9%) (Table 4). The weighted mean average of TSAT decreased by ∼50% from cord blood to venous blood in FTB neonates (down to 25.2%; 95% CI: 20.1%, 30.3%) (Figure 4A). This hypoferremic response in FTB neonates was followed by a steady increase from 21.8% (95% CI: 18.8%, 24.7%) to 44.2% (95% CI: 32.1%, 57.8%). No trend was identifiable in PTB neonates owing to the lack of data (Table 5, Figure 4B).

### Serum iron

Unlike TSAT values, serum iron concentrations in cord blood were higher in PTB neonates (46.8 μmol/L; 95% CI: 29.7, 63.8 μmol/L) than in FTB neonates (28.4 μmol/L; 95% CI: 26.0, 31.1 μmol/L) (Supplemental Figure 3). Like TSAT, a similar 50% decrease in the weighted mean average of venous blood compared with cord blood is seen in FTB (13.8 μmol/L; 95% CI: 10.8, 16.9 μmol/L) (Table 6) and PTB neonates (16.2 μmol/L; 95% CI: 15.3, 17.0 μmol/L) (Table 7). Figure 5 suggests that after the initial reduction (in the first 48 h of life), concentrations of serum iron remain consistent over the first month of life in (A) FTB and (B) PTB neonates. Serum iron was lowest between 0 and 48 h postdelivery (Tables 6, 7).

## Discussion

### Hypoferremia in FTB neonates

The weighted mean average for cord blood hepcidin was calculated using data from 11 studies. Almost all included studies reported a mean value between 11 and 41 ng/mL, apart from Kulik-Rechberger et al. (46). This study reported a much higher cord blood hepcidin value (67.9 ng/mL; 95% CI: 59.3, 76.5 ng/mL), as Supplemental Figure 1A shows. In addition, this study also recorded higher hepcidin concentrations in venous samples collected at 72 h (92.9 ng/mL; 95% CI: 83.3, 102.3 ng/mL) (46) than in those collected by Prentice et al. (99) at 77 h (55.6 ng/mL; 95% CI: 47.1, 65.5 ng/mL).

When all the data are reviewed together (Figure 3A), hepcidin increases from within the first 2–11 h of life (99) and then continues to increase ≤82 h postdelivery. At all times the hepcidin concentrations are much higher than those recorded in adults. This excess hepcidin production may provide a quick, comprehensive, and relatively long-lasting (0–3 d) hypoferremic response to aid protection during this vulnerable period (99). After the first few days, TSAT gradually increases as do serum iron concentrations, eventually reaching a plateau at ∼1 mo of age.

### Iron metabolism biomarker data gaps in the first month of life in full-term infants

Gaps in the time course of the concentrations of hepcidin and serum iron and the level of TSAT in the first month of life in full-term neonates still exist. This hinders our understanding of neonatal iron metabolism, particularly because hepcidin, TSAT, and serum iron are transient and dynamic iron parameters. At the point in which hypoferremia is believed to be maximal, publications detailing the concentrations and levels in early (<12 h) venous samples are lacking in both groups (FTB, *n* = 2; PTB, *n* = 1). Further research at this time point is required to fully elicit the strength and consistency of this response, as well as to understand the process in greater detail.

### Lack of data on preterm neonates during the first 24 h

After analysis of the current literature, the extent of the role that hypoferremia plays in neonates with a gestational age <37 wk is still unclear. This is primarily due to the limited number of publications documenting hepcidin (*n* = 5), TSAT (*n* = 6), and serum iron (*n* = 13) in the first month of life in preterm neonates. The variability between the studies is vast and further complicated by the complex, intensive, and inconsistent care of premature neonates worldwide.

**TABLE 2:** Hepcidin concentration in full-term newborns over the neonatal period^1^

						Hemoglobin,^2^ g/dL		Hepcidin, ng/mL		Standardized hepcidin,^3^ ng/mL	
Publication	Year	Location	*n*	Test type	Type of sample	Mean ± SD or mean (95% CI)	Median [IQR]	Mean (95% CI)^4^	Median ([IQR] or 95% CI or {range})^5^	Mean (95% CI)	Median ([IQR] or 95% CI or {range})^5^
Armitage et al. (115) (VPM)	2019	The Gambia	114	ELISA (Bachem)	Cord (plasma)				46.0 [31.3–55.1]		29.4 [20.0–35.2]
Armitage et al. (115) (VA)	2019	The Gambia	193	ELISA (Bachem)	Cord (plasma)		13.7 [12.4–14.6]		41.9 [26.3–56.6]		26.8 [16.8–36.1]
Basu et al. (51)	2015	India	15	ELISA (Hangzhou Eastbiopharm)^6^	Cord (serum)	16.3 ± 1.6		124.0 (115, 133)		N/A	
Briana et al. (50)	2013	Greece	104	ELISA (DRG)	Cord (serum)	17.5 ± 2.0			17.85 {4.75–69.2}		24.1 {3.1–44.2}
Cao et al. (116)	2014	USA	57	ELISA (Intrinsic)	Cord (serum)	13.7 ± 2.7		131.8 (109, 155)		41.7 (34.5, 48.9)	
Cao et al. (117)	2016	USA	98	ELISA (Intrinsic)	Cord (serum)	14.0 ± 2.8		121.5 (105, 138)		38.3 (33.2, 43.6)	
Delaney et al. (118)	2019	USA	107	ELISA (Intrinsic)	Cord (serum)	14.3 ± 2.5		92.13 (91.9, 92.3)		29.2 (29.1, 29.2)	
Dosch et al. (119)	2016	USA	47	ELISA (DRG)	Cord (plasma)	14.6 ± 1.4		13.4 (11.7, 15.1)		17.8 (15.4, 20.2)	
Garcia-Valdes et al. (57)	2015	Spain	52	ELISA (DRG)	Cord (serum)	16.9 ± 1.7		18.01 (15.1, 20.9)		24.3 (20.2, 28.4)	
Hoppe et al. (80)	2019	Sweden	15	ELISA (Bachem)	Cord (serum)				30.5 [21.7–38.8]		19.5 [13.9–24.8]
Kulik-Rechberger et al. (46)	2016	Poland	44	ELISA (DRG)	Cord (serum)			48.98 (42.9, 55.1)		67.9 (59.3, 76.5)	
			44		Venous: 72 h (serum)	16.8 ± 2.1		66.79 (60.0, 73.5)		92.9 (83.3, 102.3)	
Lee et al. (43)	2016	USA	104	ELISA (Intrinsic)	Cord (serum)	14.2 ± 2.8		87.4 (74.4, 103)		27.7 (23.6, 32.6)	
Lorenz et al. (84)	2014	Germany	100	ELISA (Intrinsic)	Cord (plasma)	15.5 ± 1.9			103.9 [61.4–149.2]		32.9 [19.6–47.1]
Prentice et al. (99)	2019	The Gambia	81	ELISA (Bachem)	Cord (serum)	14.4 (13.8, 14.9)		43.8 (36.8, 52.3)		27.9 (23.5, 33.4)	
			53		Venous: 6 h [2–11 h] (serum)^7^	17.6 (17.1, 18.2)		79.4 (68.1, 92.4)		50.7 (43.5, 58.9)	
			21		Venous: 29 h [26–34 h] (serum)^7^	19.2 (18.3, 20.0)		45.9 (36.5, 57.8)		29.3 (23.3, 36.9)	
			33		Venous: 77 h [74–82 h] (serum)^7^	17.9 (17.0, 18.7)		87.1 (73.8, 102.7)		55.6 (47.1, 65.5)	
Rehu et al. (24)	2010	Finland	116	ELISA (Intrinsic)	Cord (serum)			71.6 (60.8, 84.4)		22.8 (19.4, 26.77)	
Ru et al. (120)	2018	USA	50	ELISA (Bachem)	Cord (serum)	15.3 ± 0.4		17 (12.0, 24.2)		10.9 (7.7, 15.5)	
Słomka et al. (93)	2013	Poland	54	ELISA (DRG)	Cord (serum)				18.50 [2.75–35.13]		25 [2.8–48.4]
Young et al. (121)	2012	USA	19	ELISA (Intrinsic)	Cord (serum)	13.4 ± 3.0		61.0 (26.4, 95.6)		19.4 (8.6, 30.3)	
Weighted mean (cord)						—	—	73.2 (48.1, 98.3)	—	29.7 (21.1, 38.3)	—
Weighted mean (venous)						—	—	72.7 (48.3, 97.2)	—	61.1 (20.1, 102.0)	—
Male adults^8^ (92)									13.1 (1.4, 43.2)		
Female adults^8^ (92)									10.6 (1.4, 43.0)		
Infants^8^ (92)									11.9 (3.3, 37.7)		

1 N/A, not applicable; VA, Vitamin A study; VPM, Vaccination and Paediatric Microbiome study; —, not determined because not applicable to the calculation of weighted mean hepcidin or standardized hepcidin values.

2 Hemoglobin concentrations are provided to aid interpretation of neonatal iron status.

3 Hepcidin standardization was conducted using the linear equations documented in Supplemental Table 1.

4 Extracted SDs were converted to 95% CIs.

5 Medians ([IQRs] or 95% CIs or ranges) were not included in weighted means.

6 Values from Basu et al. (51) were not standardized because the study used the Hangzhou Eastbiopharm ELISA, which was not part of the van der Vorm et al. (89) analysis.

7 Median [IQR].

8 Reference ranges for adults (male and female) and infants are displayed for comparison (92).

**TABLE 3:** Hepcidin concentration in preterm newborns over the neonatal period^1^

							Hemoglobin,^2^ g/dL		Hepcidin, ng/mL		Standardized hepcidin,^3^ ng/mL	
Publication	Year	Location	*n*	Test type	Type of sample	Study group	Mean ± SD or mean (95% CI)	Median [IQR]	Mean (95% CI)^4^	Median ([IQR] or 95% CI)^5^	Mean (95% CI)^4^	Median ([IQR] or 95% CI)^5^
Delaney et al. (118)	2019	USA	126	ELISA (Bachem)	Cord (serum)		15.3 ± 2.3		13.78 (13.6, 14.0)		8.8 (8.7, 9.0)	
Ichinomiya et al. (82)	2017	Japan	92	Mass Spec (MCProt)	Cord (serum)					7.3 [2.85–16.38]		*
Lorenz et al. (84)	2014	Germany	40	ELISA (Intrinsic)	Cord (plasma)	24–29 wk		16.8 [15.1–18.0]		26.9 [13.5–63.1]		8.7 [4.5–20.1]
			81	ELISA (Intrinsic)	Cord (plasma)	30–36 wk	16.1 ± 2.2			45.9 [24.7–74.5]		14.7 [8.0–23.7]
Ru et al. (120)	2018	USA	92	ELISA (Bachem)	Cord (serum)		15.1 ± 0.3		12.1 (9.2, 15.7)		7.8 (5.9, 10.1)	
Uijterschout et al. (100)	2016	Netherlands	85	ELISA (Bachem)	Venous: 168 h (serum)			16.5 [12.0–21.5]	69.6 (14.6, 180.1)		44.4 (9.4, 114.8)	
Weighted mean (cord)					—	—	13.1 (2.4, 23.7)	—	8.4 (2.0, 14.7)	—		
Weighted mean (venous)					—	—	69.6 (14.6, 180.1)	—	44.4 (9.4, 114.8)	—		
Male adults^6^ (92)										13.1 (1.4, 43.2)		
Female adults^6^ (92)										10.6 (1.4, 43.0)		
Infants^6^ (92)										11.9 (3.3, 37.7)		

1 *Ichinomiya et al. (82) was not standardized because the study used an MS-based method that was not part of the van der Vorm et al. (89) analysis. —, not determined because not applicable to the calculation of weighted mean hepcidin or standardized hepcidin values.

2 Hemoglobin concentrations are provided to aid interpretation of neonatal iron status.

3 Hepcidin standardization was conducted using the linear equations documented in Supplemental Table 1.

4 Extracted SDs were converted to 95% CIs.

5 Medians ([IQRs] or 95% CIs) were not included in weighted means.

6 Reference ranges for adults (male and female) and infants are displayed for comparison (92).

Data analysis of the retrieved publications suggests that preterm neonates have lower cord hepcidin than full-term neonates, or infants and healthy adults. Weighted cord mean values are 250% higher in full-term (29.7 ng/mL; 95% CI: 21.1, 38.3 ng/mL) neonates than in preterm (8.4 ng/mL; 95% CI: 2.0, 14.7 ng/mL) neonates. We speculate that this could be due to very early preterm neonates (<30 weeks of gestation) possessing circulatory monocytes with decreased surface expression of toll-like receptor 4 (TLR4), lower mRNA expression of TLR4, and reduced cytokine production (122). An effect on the production of IL-6 at delivery might then lead to a reduced ability to stimulate hepcidin expression as suggested in full-term infants.

Our analysis proposes that peripheral venous hepcidin values in preterm neonates increase to 44 ng/mL at 168 h. However, decreases in TSAT between the cord and venous samples are not observed (36.5%–45.6%). We propose that this is due to a lack of data on TSAT levels in preterm neonates over the first hours of life, potentially due to the complex ethical questions around bleeding preterm neonates so early in postnatal life. This results in the collection of skewed data, focusing only on later time points in the first month of life.

### Limitations

The aim of this state-of-the-art review was to evaluate our current knowledge on neonatal iron homeostasis in preterm and full-term neonates. As a result of the dearth of publications detailing the parameters of interest during this period, our review has several limitations discussed below. First, we were unable to stratify by geographical location. Many studies do not stratify their study groups by gestational age (preterm: <37 wk, full-term: ≥37 wk). Subsequently, we have had to assign each study group or population by the mean gestational age. This will result in a reduction of any natural variation potentially caused by gestational age between the reviewed populations. This is also the case with respect to birth weight and hemoglobin concentration.

Similarly, the studies on preterm neonates are made up of multiple small sample size subgroups with different gestational ages. Owing to the lack of preterm studies, we have had to combine these study groups to formulate weighted means and figures. This in itself could distort the impact of gestational age on our results, because data from the very early preterm newborns are combined with those from the late preterm neonates.

The retrieval of gestational age was a crucial aspect of the search strategy; however, few studies documented the method used. There are large differences in the accuracy of different techniques (123).

Post hoc standardization of different hepcidin ELISA kits has, to our knowledge, never been completed before with retrospective data. However, care should be given to the accuracy of the standardized values, because standardization was only possible for DRG, Bachem, and Intrinsic Lifesciences ELISA test kits. Studies that used alternative methods (124) were not included in summary statistics.

**TABLE 4:** TSAT in full-term newborns over the neonatal period^1^

					Hemoglobin,^2^ g/dL		TSAT, %	
Publication	Year	Location	*n*	Type of sample	Mean ± SD or mean (95% CI)	Median ([IQR] or 95% CI or {range})	Mean (95% CI)^3^	Median ([IQR] or 95% CI or {range})^4^
Al-Tawil et al. (102)	2012	Egypt	90	Venous: 24 h	19.6 ± 3.8		25.0 (24.6, 25.4)	
Ali et al. (125)	2016	USA	64	Cord			59.2 (53.9, 64.5)	
Andersson et al. (103)	2011	Sweden	162	Venous: 48 h	18.9 ± 1.7		23.0 (21.9, 24.1)	
Balogh et al. (94)	2007	Hungary	20	Cord		15.95 {13.4–20.7}		60.5 {14–90}
			20	Venous: 39 h (18–114 h)^5^		17.5 {13.8–20.9}		22.5 {11–42}
Basu et al. (51)	2015	India	15	Cord	16.3 ± 1.6		61.8 (54.7, 68.9)	
El-Farrash et al. (126)	2012	Egypt	30	Cord	17.7 ± 1.4		49.5 (42.5, 56.5)	
Ervasti et al. (127)	2007	Finland	199	Cord	15.9 ± 1.5		55.0 (52.4, 57.6)	
Hågå (128)	1980	Norway	21	Cord			55.0 (33.8, 76.2)	
Kalem et al. (129)	2019	Turkey	380	Cord			55.87 (54.8, 56.9)	
Kelly et al. (41)	1978	Scotland	115	Cord			58.8 (55.6, 62.0)	
Kitajima et al. (104)	2011	Japan	8	Cord				15.1 {8.3–27.5}
			8	Venous: 720 h				44.2 {32.1–57.8}
Kleven et al. (130)	2007	USA	26	Cord	15.7 ± 1.0		42.0 (32.4, 51.6)	
Mashako et al. (131)	1991	DRC	166	Cord			32.3 (30.1, 34.5)	
Milman et al. (39)	1987	Denmark	74	Cord		16.1 (14.3, 18.2)		48 (32, 71)
			47	Venous: 120 h				33 (21, 48)
Prentice et al. (99)	2019	The Gambia	81	Cord	14.4 (13.8, 14.9)		47.6 (43.7, 51.5)	
			53	Venous: 6 h [2–11 h]^6^	17.6 (17.1, 18.2)		24.4 (21.2, 27.6)	
			21	Venous: 29 h [26–34 h]^6^	19.2 (18.3, 20.0)		21.8 (18.8, 24.7)	
			33	Venous: 77 h [74–82 h]^6^	17.9 (17.0, 18.7)		30.9 (26.9, 34.8)	
Puolakka et al. (54)	1980	Finland	47	Cord	15.1 ± 1.2		53 (49, 57)	
Rehu et al. (24)	2010	Finland	116	Cord			50.6 (44.5, 57.5)	
Rios et al. (132)	1975	USA	20	Cord	16.1 ± 1.5		61.2 (55.9, 66.5)	
Słomka et al. (93)	2013	Poland	49	Cord				58.1 [51.7–73.6]
Yamada and Leone (105)	2014	Brazil	21	Cord	16.0 ± 1.8		47.7 (40.2, 55.2)	
			21	Venous: 720 h	12.0 ± 2.0		39.8 (34.7, 44.9)	
Weighted mean (cord)					—	—	51.7 (46.5, 56.9)	—
Weighted mean (venous)					—	—	25.2 (20.1, 30.3)	—
Adults^7^ (91)								23.5 (12.0, 38.8)
Infants^7^ (91)								19.4 (8.2, 32.9)

1 TSAT, transferrin saturation; —, not determined because not applicable to the calculation of weighted mean hepcidin or standardized hepcidin values.

2 Hemoglobin concentrations are provided to aid interpretation of neonatal iron status.

3 Extracted SDs were converted to 95% CIs.

4 Medians ([IQRs] or 95% CIs) were not included in weighted means.

5 Median (minimum–maximum range).

6 Median [IQR].

7 Reference ranges for adults and infants are taken from the NHANES, 1999–2000 (91).

**TABLE 5:** TSAT in preterm newborns over the neonatal period^1^

						Hemoglobin,^2^ g/dL		TSAT, %	
Publication	Year	Location	*n*	Type of sample	Study group	Mean ± SD	Median ([IQR] or 95% CI or {range})	Mean (95% CI)^3^	Median ([IQR] or 95% CI or {range})^4^
Celik et al. (107)	2015	Turkey	42	Venous: 648 h (288–1872 h)^5^		13.4 ± 4.0		46.5 (41.2, 51.8)	
Hågå (128)	1980	Norway	23	Cord	AGA group^6^			48.0 (39.8, 56.2)	
			6	Cord	SGA group^7^			41.0 (23.4, 58.6)	
Ichinomiya et al. (82)	2017	Japan	92	Cord					87.2 [68.3–100]
Kitajima et al. (104)	2011	Japan	13	Cord					64.3 {15.8–88.9}
			13	Venous: 720 h					33.2 {17.1–79.5}
Lackmann et al. (106)	1998	Germany	15	Venous: <1 h^8^	<32 wk				39 (5, 83)
			22	Venous: <1 h^8^	33–34 wk				36 (7, 87)
			26	Venous: <1 h^8^	35–36 wk				31 (13, 60)
Yamada and Leone (105)	2014	Brazil	25	Cord		15.7 ± 1.8		24.8 (18.5, 31.1)	
			25	Venous: 720 h		10.8 ± 1.8		44.1 (37.3, 50.9)	
Weighted mean (cord)						—	—	36.5 (0.8, 72.1)	—
Weighted mean (venous)						—	—	45.6 (30.4, 60.9)	—
Adults^9^ (91)									23.5 (12.0, 38.8)
Infants^9^ (91)									19.4 (8.2, 32.9)

1 AGA, appropriate for gestational age; SGA, small for gestational age; TSAT, transferrin saturation; —, not determined because not applicable to the calculation of weighted mean hepcidin or standardized hepcidin values.

2 Hemoglobin concentrations are provided to aid interpretation of neonatal iron status.

3 Extracted SDs were converted to 95% CIs.

4 Medians ([IQRs] or 95% CIs) were not included in weighted means.

5 Median (minimum–maximum range).

6 AGA group of Hågå (128) can be identified in Supplemental Figure 2B.

7 SGA group of Hågå (128) can be identified in Supplemental Figure 2B.

8 Minimum–maximum range.

9 Reference ranges for adults and infants are taken from the NHANES, 1999–2000 (91).

An essential criterion of inclusion in this publication was that all neonatal data came from healthy newborns. However, documentation of labor practices (including mode of delivery) and postnatal care, along with postnatal medication, lack detail in the publications retrieved. Vaginal delivery is occasionally referred to as the method of delivery; however, the use of inflammation-inducing forceps, cesarean delivery, or vacuum delivery is not consistently reported in each publication.

**TABLE 6:** Serum iron concentration in full-term newborns over the neonatal period^1^

					Hemoglobin,^2^ g/dL		Serum iron, μmol/L	
Publication	Year	Location	*n*	Type of sample	Mean ± SD or mean (95% CI)	Median ([IQR] or 95% CI or {range})	Mean (95% CI or {range})^3^	Median ([IQR] or 95% CI or {range})^4^
Ahlsten et al. (133)	1989	Sweden	20	Cord			38.0 (34.9, 41.1)	
Ali et al. (125)	2016	USA	64	Cord			26.8 (24.4, 29.2)	
Amarnath et al. (134)	1989	USA	15	Cord			24.1 (21.0, 27.2)	
Andersson et al. (103)	2011	Sweden	162	Venous: 48 h	18.9 ± 1.7		9.9 (9.5, 10.3)	
Armitage et al. (115) (VA)	2019	The Gambia	193	Cord		13.7 [12.4–14.6]		18.8 [15.4–22.3]
Armitage et al. (115) (VPM)	2019	The Gambia	114	Cord				16.0 [12.7–18.7]
Awadallah et al. (32)	2004	Jordan	92	Cord	15.7 ± 2.6		20.7 (20.1, 21.3)	
Balogh et al. (94)	2007	Hungary	20	Cord		15.95 {13.4–20.7}		25.5 {8–43}
			20	Venous: 39 h (18–114 h)^5^		17.5 {13.8–20.9}		9.5 {5–20}
Bastida et al. (135)	2000	Spain	70	Cord			41.5 (38.3, 44.7)	
Basu et al. (51)	2015	India	15	Cord	16.3 ± 1.6		23.8 (22.2, 25.4)	
Basu et al. (124)	2015	India	142	Cord	16.3 ± 1.5		26.5 (25.5, 27.5)	
Bermúdez et al. (136)	2015	Spain	30	Cord			6.26 (5.37, 7.15)	
Briana et al. (50)	2013	Greece	104	Cord	17.5 ± 2.0		24.14 (22.4, 25.9)	
Busarira et al. (137)	2019	Libya	126	Cord	13.7 ± 1.4		23.69 (23.5, 23.9)	
Cao et al. (117)	2016	USA	68	Cord	14.0 ± 2.8		39.73 (35.0, 44.4)	
Chong et al. (138)	1984	UK	20	Cord	15.8 ± 2.1		41.1 (29.6, 52.6)	
Delaney et al. (118)	2019	USA	101	Cord	14.3 ± 2.5		40.8 (37.3, 44.3)	
El-Farrash et al. (126)	2012	Egypt	30	Cord	17.7 ± 1.4		28.29 (25.6, 31.0)	
Ertekin et al. (139)	2015	Turkey	76	Cord	15.2 ± 1.8		26.1 (24.1, 28.1)	
Ervasti et al. (127)	2007	Finland	199	Cord	15.9 ± 1.5		27.4 (26.3, 28.5)	
Gruccio et al. (140)	2014	Argentina	99	Cord			27.03 (25.7, 28.4)	
Hågå (128)	1980	Norway	21	Cord			27.1 (24.2, 30.0)	
Kelly et al. (41)	1978	Scotland	115	Cord			27.0 (25.6, 28.4)	
Kleven et al. (130)	2007	USA	26	Cord	15.7 ± 1.0		44.1 (32.2, 56.0)	
Kocyłowski et al. (38)	2018	Poland	64	Cord			35.1 (33.2, 37.0)^6^	
Lao et al. (40)	1991	Hong Kong	77	Cord	15.6 ± 1.9		35.8 (32.7, 38.9)	
Lee et al. (34)	2006	South Korea	19	Cord			31.3 (28.4, 34.2)	
Lee et al. (43)	2016	USA	82	Cord	14.2 ± 2.8		35.4 (32.0, 39.2)	
Mezdoud et al. (141)	2017	Algeria	97	Cord	14.8 ± 1.8		20.1 (19.0, 21.3)	
Milman et al. (39)	1987	Denmark	74	Cord		16.1 (14.3, 18.2)		28 (19, 39)
			47	Venous: 120 h				19 (13, 31)
Mukhopadhyay et al. (142)	2012	India	50	Cord	15.8 ± 1.4		29 (25.8, 32.2)	
Murata et al. (143)	1989	Japan	45	Cord	14.8 ± 1.5		28.5 (26.7, 30.3)	
de Cássia Carvalho Oliveira et al. (144)	2014	Brazil	144	Cord	14.7 ± 1.5		24.6 (23.5, 25.7)	
Ozkiraz et al. (113)	2011	Turkey	16	Venous: 216 h (96–336 h)^5^	14.0 ± 1.3		16.4 (13.8, 19.0)	
Patidar et al. (108)	2013	India	50	Venous: 8 h (1–23 h)^5^	15.0 ± 2.0		19.4 (17.2, 21.6)	
Prentice et al. (99)	2019	The Gambia	81	Cord	14.4 (13.8, 14.9)		24.7 (22.5, 26.9)	
			53	Venous: 6 h [2–11 h]^7^	17.6 (17.1, 18.2)		13.6 (12.0, 15.2)	
			21	Venous: 29 h [26–34 h]^7^	19.2 (18.3, 20.0)		11.6 (10.1, 13.1)	
			33	Venous: 77 h [74–82 h]^7^	17.9 (17.0, 18.7)		14.5 (13.1, 16.0)	
Puolakka et al. (54)	1980	Finland	47	Cord	15.1 ± 1.2		28.8 (26.2, 31.4)	
Rios et al. (132)	1975	USA	20	Cord	16.1 ± 1.5		0.026 (0.023, 0.028)	
Ru et al. (120)	2018	USA	49	Cord	15.3 ± 0.4		48.3 (39.3, 59.1)	
Słomka et al. (93)	2013	Poland	49	Cord			22.4 (20.5, 24.3)	
Sweet et al. (145)	2001	UK	68	Cord	16.1 ± 1.7		26.0 (24.2, 27.8)	
Szabó et al. (109)	2001	Hungary	10	Cord			23.2 (16.3, 30.1)	
			10	Venous: 47 ± 6 h^8^			7.20 (6.15, 8.25)	
Tiker et al. (111)	2006	Turkey	16	Venous: 209 h (96–288 h)^9^		13.9 {12.2–17.1}	19.9 {12.7–28.4}	
Tsuzuki et al. (110)	2013	Japan	30	Cord			31.1 (27.3, 34.9)	
			30	Venous: 120 h			19.5 (16.4, 22.6)	
Yamada and Leone (105)	2014	Brazil	21	Cord	16.0 ± 1.4		23.9 (19.4, 28.4)	
			21	Venous: 720 h	12.0 ± 2.0		16.7 (14.9, 18.5)	
Yapakçı et al. (112)	2009	Turkey	16	Venous: 211 ± 46 h^8^	14.0 ± 1.4		19.9 (17.9, 21.9)	
Weighted mean (cord)					—	—	28.4 (26.0, 31.1)	—
Weighted mean (venous)					—	—	13.8 (10.8, 16.9)	—
Adults^10^ (91)								15.2 (8.1, 24.5)
Infants^10^ (91)								12.5 (5.5, 20.6)

1 VA, Vitamin A study; VPM, Vaccination and Paediatric Microbiome study; —, not determined because not applicable to the calculation of weighted mean hepcidin or standardized hepcidin values.

2 Hemoglobin concentrations are provided to aid interpretation of neonatal iron status.

3 Extracted SDs were converted to 95% CIs.

4 Medians ([IQRs] or 95% CIs) were not included in weighted means.

5 Median (minimum–maximum range).

6 Umbilical cord vein and artery serum means were combined.

7 Median [IQR].

8 Mean ± SD.

9 Mean (minimum-maximum range).

10 Reference ranges for adults and infants are taken from the NHANES, 1999–2000 (91).

**TABLE 7:** Serum iron concentration in preterm newborns over the neonatal period^1^

						Hemoglobin,^2^ g/dL		Serum iron, μmol/L	
Publication	Year	Location	*n*	Type of sample(cord or venous)	Study group	Mean ± SD	Median ([IQR] or 95% CI or {range})	Mean (95% CI or {range})^3^	Median ([IQR] or 95% CI)^4^
Celik et al. (107)	2015	Turkey	42	Venous: 648 h (288–1872 h)^5^		13.4 ± 4.0		15.6 (13.3, 17.9)	
Delaney et al. (118)	2019	USA	123	Cord		15.3 ± 2.3		53.1 (49.8, 56.4)	
Hågå (128)	1980	Norway	24	Cord	AGA group^6^			16.8 (13.2, 20.4)	
			7	Cord	SGA group^7^			18.3 (11.2, 25.4)	
Ichinomiya et al. (82)	2017	Japan	92	Cord					23.27 [15.2–32.4]
Lackmann et al. (106)	1998	Germany	15	Venous: <1 h^8^	<32 wk				14 (2, 41)
			22	Venous: <1 h^8^	33–34 wk				12 (2, 32)
			26	Venous: <1 h^8^	35–36 wk				15 (6, 28)
Ru et al. (120)	2018	USA	91	Cord	^9^	15.1 ± 0.3		73.4 (57.3, 93.1)	
Ru et al. (49)	2018	USA	140	Cord	^10^	15.3 ± 2.1		53.7 (50.1, 60.8)	
Schiza et al. (114)	2007	Greece	181	Venous: 336 h		12 ± 1		16.1 (15.5, 16.7)	
Sweet et al. (145)	2001	UK	50	Cord	30–36 wk^11^	15.8 ± 2.1		20.8 (18.4, 23.2)	
			26	Cord	24–29 wk^12^	16.3 ± 1.6		17.4 (13.3, 21.5)	
Tiker et al. (111)	2006	Turkey	14	Venous: 67 h (24–144 h)^13^	26–32 wk		14.9 {10.5–18.5}	15.81 {4.83–33.48}	
			12	Venous: 65 h (24–96 h)^13^	33–36 wk		15.7 {13.3–21.5}	19.26 {6.8–39.2}	
Tsuzuki et al. (110)	2013	Japan	14	Cord				27.5 (21.9, 33.1)	
				Venous: 120 h				16.47 (13.1, 19.8)	
Yamada and Leone (105)	2014	Brazil	25	Cord		15.7 ± 1.8		8.8 (6.97, 10.6)	
				Venous: 720 h		10.8 ± 1.8		15.54 (14.1, 17.0)	
Yapakçı et al. (112)	2009	Turkey	17	Venous: 336 h (96–720 h)^13^		12.7 ± 2.2		17.22 (14.2, 20.3)	
Weighted mean (cord)						—	—	46.8 (29.7, 63.8)	—
Weighted mean (venous)						—	—	16.2 (15.3, 17.0)	—
Adults^14^ (91)									15.2 (8.1, 24.5)
Infants^14^ (91)									12.5 (5.5, 20.6)

1 AGA, appropriate for gestational age; SGA, small for gestational age; —, not determined because not applicable to the calculation of weighted mean hepcidin or standardized hepcidin values.

2 Hemoglobin concentrations are provided to aid interpretation of neonatal iron status.

3 Extracted SDs were converted to 95% CIs.

4 Medians ([IQRs] or 95% CIs) were not included in weighted means.

5 Median (minimum–maximum range).

6 AGA group of Hågå (128) can be identified in Supplemental Figure 3B.

7 SGA group of Hågå (128) can be identified in Supplemental Figure 3B.

8 Minimum-maximum range.

9 Ru et al. (120) can be identified in Supplemental Figure 3B.

10 Ru et al. (49) can be identified in Supplemental Figure 3B.

11 30–36 wk group of Sweet et al. (145) can be identified in Supplemental Figure 3B.

12 24–29 wk group of Sweet et al. (145) can be identified in Supplemental Figure 3B.

13 Mean (minimum–maximum range).

14 Reference ranges for adults and infants are taken from the NHANES, 1999–2000 (91).

### Conclusion

Currently available data suggest that hepcidin and serum iron concentrations and TSAT levels for adults and infants are much lower than those found in cord blood and venous blood from neonates during the first month of life. We have strengthened the evidence that FTB neonates possess the ability to produce a hepcidin-mediated hypoferremic response postdelivery. Whether this mechanism is found in PTB neonates is still unclear. This is predominately due to the lack of studies on healthy preterm neonates during the first hours of life. If premature or low-birth-weight neonates are unable to mount a hypoferremic response, this could enhance their risk of early neonatal infections. Conversely, if the hypoferremic response is seen in both preterm and full-term neonates, it will further support the hypothesis that regulation of iron distribution plays a fundamental role as an innate mechanism of protection against infection.

In summary, serum hepcidin is likely triggered by the inflammatory effect of labor and delivery. We suggest that this intrinsic mechanism of protection protects newborns with immature immune systems as they transition from a semi-allogeneic, protected fetal setting to a microbe-rich extrauterine environment (146, 147). Hepcidin-induced hypoferremia then potentially provides a broad-action innate bacteriostatic action against invading micro-organisms, when physiological adaption to postnatal life is so critical for survival.

## Supplementary Material

nzaa104_Supplemental_File

## Notes

Supported by Bill & Melinda Gates Foundation research grant OPP1152353 (to CC). The funding agency had no role in the design of this state-of-the-art review, and did not have any in the collection, management, analyses, or interpretation of the data nor in the preparation, review, or approval of the manuscript. The Nutrition Theme of the Medical Research Council (MRC) Unit The Gambia at the London School of Hygiene & Tropical Medicine is supported by core funding MC-A760-5QX00 to the MRC Unit The Gambia/MRC International Nutrition Group by the UK MRC and the UK Department for International Development (DFID) under the MRC/DFID Concordat agreement.

Author disclosures: The authors report no conflicts of interest.

Supplemental Table 1 and Supplemental Figures 1–3 are available from the “Supplementary data” link in the online posting of the article and from the same link in the online table of contents at https://academic.oup.com/cdn/.

Abbreviations used: CRP, C-reactive protein; EIA, enzyme immunoassay; FTB, full-term-birth; PTB, preterm-birth; TFR1, transferrin receptor 1; TLR4, toll-like receptor 4; TSAT, transferrin saturation.
