# Supplementary material for: Hepcidin, Serum Iron, and Transferrin Saturation in Full-Term and Premature Infants during the First Month of Life: A State-of-the-Art Review of Existing Evidence in Humans
Source: Curr Dev Nutr. 2020 Jun 17;4(8):nzaa104. doi: 10.1093/cdn/nzaa104 (PMC7413980; doi:10.1093/cdn/nzaa104)
Supplement: nzaa104_Supplemental_File [file nzaa104_supplemental_file.docx]

**Hepcidin, serum iron and transferrin saturation in full term and premature infants during the first month of life: A state-of-the-art review of existing evidence in humans. Cross JH et al**  **“Online Supplementary Material”**

**SUPPLEMENTARY METHODS**

**Supplementary Table 1 - Post-hoc hepcidin standardization**

| **Test ID** | **Kit** | **Slope (95% CI)** | **Intercept (95% CI)** |
| --- | --- | --- | --- |
| IC-4 | Bachem Hepcidin-25 | 1.569 (1.523-1.614) | -0.10 (-1.95 to 1.85) |
| IC-5 | Instrinsic Lifesciences Hepcidin IDx | 3.184 (3.086-3.281) | -0.85 (-2.91 to 1.21) |
| IC-6 | DRG Hepcidin-25 | 0.711 (0.674-0.748) | 0.73 (-0.05 to 1.52) |

**Supplementary Table 1:** Previously documented regression relationships used to conduct post-hoc hepcidin standardization. These regression relationships were extract from **Table 2** of van der Vorm *et al*.(1)

**SUPPLEMENTARY FIGURES**

**Supplementary Figure 1: Standardized hepcidin concentration (ng/mL) in cord blood.**

**Supplementary Figure 2: Transferrin saturation (%) in cord blood.**

**Supplementary Figure 3: Serum iron concentration (μmol/L) in cord blood**

**SUPPLEMENTARY FIGURE LEGENDS**

**Supplementary Figure 1: Standardized hepcidin (ng/mL) in cord blood:** (**A**) full term neonates, (**B**) preterm neonates. Standardized means from each publication are plotted with error bars showing 95% confidence intervals. Dashed line shows the weighted mean of all publications found in the figure. Median values were not included in this figure.

**Supplementary Figure 2: Transferrin saturation (%) in cord blood:** (**A**) full term neonates, (**B**) preterm neonates. Means from each publication are plotted with error bars showing 95% confidence intervals. Dashed line shows the weighted mean of all publications found in the figure. ^a^ shows Haga *et al*, AGA group.(2) ^b^ shows Haga *et al* SGA group.(2) Median values were not included in this figure.

**Supplementary Figure 3: Serum iron (μmol/L) in cord blood:** (**A**) full term neonates, (**B**) preterm neonates. Means from each publication are plotted with error bars showing 95% confidence intervals. Dashed line shows the weighted mean of all publications found in the figure. ^c^ shows AGA neonates in Haga *et al.*(2) ^d^ shows SGA neonates in Haga *et al.*(2) Ru *et al*, 2018(3) is referenced as ^e^. Ru *et al*, 2018(4) is referenced as ^f^. ^g^ shows 30-36 wks neonates in Sweet *et al.*(5) ^h^ shows 24-29 wks neonates in Sweet *et al.*(5) Median values were not included in this figure.

**SUPPLEMENTARY REFERENCES**

1. van der Vorm LN, Hendriks JCM, Laarakkers CM, Klaver S, Armitage AE, Bamberg A, Geurts-Moespot AJ, Girelli D, Herkert M, Itkonen O, et al. Toward Worldwide Hepcidin Assay Harmonization: Identification of a Commutable Secondary Reference Material. Clin Chem [Internet]. Clinical Chemistry; 2016 [cited 2019 Sep 20];62:993–1001. Available from: http://www.ncbi.nlm.nih.gov/pubmed/27173010

2. Hågå P. Plasma ferritin concentrations in preterm infants in cord blood and during the early anaemia of prematurity. Acta Paediatr Scand [Internet]. 1980 [cited 2019 May 17];69:637–41. Available from: http://www.ncbi.nlm.nih.gov/pubmed/7234384

3. Ru Y, Pressman EK, Guillet R, Katzman PJ, Bacak SJ, O’Brien KO. Predictors of anemia and iron status at birth in neonates born to women carrying multiple fetuses. Pediatr Res [Internet]. 2018 [cited 2019 Apr 5];84:199–204. Available from: http://www.nature.com/articles/s41390-018-0044-6

4. Ru Y, Pressman EK, Guillet R, Katzman PJ, Vermeylen F, O’Brien KO. Umbilical Cord Hepcidin Concentrations Are Positively Associated with the Variance in Iron Status among Multiple Birth Neonates. J Nutr [Internet]. Oxford University Press; 2018 [cited 2018 Dec 29];148:1716–22. Available from: https://academic.oup.com/jn/article/148/11/1716/5105880

5. Sweet DG, Savage GA, Tubman R, Lappin TRJ, Halliday HL. Cord blood transferrin receptors to assess fetal iron status. Arch Dis Child Fetal Neonatal Ed [Internet]. England; 2001 [cited 2019 Apr 5];85:F46-8. Available from: http://ovidsp.ovid.com/ovidweb.cgi?T=JS&PAGE=reference&D=med4&NEWS=N&AN=11420322
